# Comparative Efficacy of First-Line Immune-Based Combination Therapies in Metastatic Renal Cell Carcinoma: A Systematic Review and Network Meta-Analysis

**DOI:** 10.3390/cancers12061673

**Published:** 2020-06-24

**Authors:** Reza Elaidi, Letuan Phan, Delphine Borchiellini, Philippe Barthelemy, Alain Ravaud, Stéphane Oudard, Yann Vano

**Affiliations:** 1 ARTIC (Association pour la Recherche sur les Thérapeutiques Innovantes en Cancérologie), Hôpital Européen Georges Pompidou, 75015 Paris, France; letuanp@gmail.com; 2 Department of Medical Oncology, Centre Antoine-Lacassagne, 06100 Nice, France; delphine.borchiellini@nice.unicancer.fr; 3 Department of Medical Oncology, Hôpital civil, 67091 Strasbourg, France; p.barthelemy@icans.eu; 4 Department of Medical Oncology, Hôpital St Andre, 33000 Bordeaux, France; alain.ravaud@chu-bordeaux.fr; 5 Department of Medical Oncology, Hôpital Européen Georges Pompidou, 75015 Paris, France; stephane.oudard@aphp.fr (S.O.); yann.vano@aphp.fr (Y.V.)

**Keywords:** metastatic renal cell carcinoma, immune-based combination therapies, network meta-analysis

## Abstract

Three drug combinations, ipilimumab-nivolumab (Ipi-Nivo), pembrolizumab-axitinib (Pembro-Axi), and avelumab-axitinib (Ave-Axi), have received regulatory approval in the USA and Europe for the treatment of metastatic renal cell carcinoma with clear cell component (mRCC). However, no head-to-head comparison data are available to identify the best option. Therefore, we aimed to compare these new treatments in a first-line setting. We conducted a systematic search in PubMed, the Cochrane Library, and clinicaltrials.gov for any randomized controlled trials of treatment-naïve patients with mRCC, from January 2015 to October 2019. The process was performed according to PRISMA guidelines. We performed a Bayesian network meta-analysis with two different approaches, a contrast-based model comparing HRs and ORs between studies and arm-based using parametric modeling. The outcomes for the analysis were overall survival, progression-free survival (PFS), and objective response rate. Our search identified 3 published phase 3 randomized clinical trials (2835 patients). In the contrast-based model, Ave-Axi (SUCRA = 83%) and Pembro-Axi (SUCRA = 80%) exhibited the best ranking probabilities for PFS. For overall survival (OS), Pembro-Axi (SUCRA = 96%) was the most preferable option against Ave-Axi and Ipi-Nivo. Objective response rate analysis showed Ave-Axi as the best (SUCRA: 94%) and Pembro-Axi as the second best option. In the parametric models, the risk of progression was comparable for Ave-Axi and Ipi-Nivo, whereas Pembro-Axi exhibited a lower risk during the first 6 months of treatment and a higher risk afterwards. Furthermore, Pembro-Axi exhibited a net advantage in terms of OS over the two other regimens, while Ave-Axi was the least preferable option. Overall evidence suggests that pembrolizumab plus axitinib seems to have a slight advantage over the other two combinations.

## 1. Background

Over the past few years, the treatment for metastatic renal cell carcinoma with clear cell component (mRCC) has drastically changed with the introduction of targeted therapy, immunotherapy, and a better understanding of RCC biology [1,2,3,4]. So far, the first-line and second-line systematic therapies for mRCC have been mainly composed of agents targeting the vascular endothelial growth factor receptor (VEGFR) and inhibiting the mammalian target of rapamycin (mTOR), with the last in class being axitinib and cabozantinib [5,6]. Currently, drug development in mRCC focuses on immune checkpoint inhibitors (ICIs), targeting programmed cell death 1 (PD-1), programmed cell death-ligand 1 (PD-L1) pathway, or cytotoxic T-lymphocyte-associated protein 4 (CTLA-4) [4,7]. Four combinations have demonstrated either progression-free survival (PFS) or overall survival (OS) improvements over the VEGFR tyrosine kinase inhibitor (TKI) standard of care (SOC) sunitinib in the first-line setting for advanced or metastatic RCC with clear cell component: nivolumab (anti-PD-1) plus ipilimumab (anti-CTLA-4) [8], pembrolizumab (anti-PD-1) plus axitinib (VEGFR-TKI) [9], avelumab (anti-PD-L1) plus axitinib [10], and atezolizumab (anti-PD-L 1) plus bevacizumab (anti-VEGF) [11].

The shift in systemic therapy of mRCC has just begun, and phase 3 results with these new available combinations raise many questions that need to be addressed in order to make better use of them in clinical practice. In addition, we still lack predictive biomarkers and prognostic characteristics in patients or the disease to guide treatment allocation. Results of these phase 3 trials should be interpreted in the context of the International Metastatic RCC Database Consortium (IMDC) risk classification, which has proven its utility since the targeted therapy era [12,13].

Comparison between these therapeutic options is one of the main concerns for clinicians and patients [14]. However, since no clinical trial has provided any head-to-head comparison data of these combinations, we conducted a network meta-analysis (NMA) to indirectly compare their efficacies in terms of progression-free survival (PFS), overall survival (OS), and objective response rate (ORR) in the first-line setting for patients with mRCC.

## 2. Methods

### 2.1. Search Strategy and Selection Criteria

We specifically focused on randomized controlled trials (RCTs), including naïve-treatment patients with mRCC with clear cell component who received one of the combinations involving ICIs in the first-line setting, with no patient restrictions on PD-L1 or the IMDC subgroup. The study was conducted based on PRISMA extended guidelines for network meta-analysis [15]. We performed a systematic literature search for articles or abstracts in PubMed, the Cochrane Library, clinicaltrials.gov, and ESMO or ASCO congress, from January 2015 to August 2019 (full search strategy detailed in File S1). References of relevant articles were checked to ensure that no combinations with ICIs were missed. If several data reports were available from the same trial, we retained the latest updated source. The outcomes were PFS, OS, and ORR in the intention-to-treat (ITT) population, and per IMDC subgroup if available. For PFS and OS, hazard ratios (HRs) with 95% confidence intervals (CIs), and their corresponding Kaplan–Meier curves (when available) were retained for our study.

The whole process of trial selection, full-text screening, and data extraction was performed by two investigators (R-E, L-P) independently; if disagreement occurred, it was resolved by discussion with other investigators. For all selected studies, risk of bias was assessed with the Cochrane Handbook tool [16].

### 2.2. Statistical Analysis

We used two different approaches: a contrast-based method comparing the relative treatment effect in the intention-to-treat population as well as in the IMDC subgroups, and an arm-based method using Kaplan–Meier curves to estimate the parametric survival model (in the ITT population only). We performed both fixed-effect and random-effect models for the contrast-based approach. To assess which treatment was likely to be the best option, we used rank probabilities and the surface under the cumulative ranking curve (SUCRA) [17] in the contrast-based NMA model and assessed time-dependent HRs derived from the arm-based NMA approach. Additionally, an exploratory analysis of the PFS of sarcomatoid carcinoma patients was performed to investigate the recently observed benefit of these combinations in this subpopulation.

#### 2.2.1. Contrast-Based Approach

This approach focused on relative effects using HR on a log scale to run an NMA model as described in Dias 2013 [18].

#### 2.2.2. Arm-Based Approach

To circumvent the apparent violation of the proportional hazard assumption of the Cox model in the published PFS Kaplan–Meier curves of the CheckMate 214 study [4], we also considered a method relying on time-dependent HRs. We used fractional polynomials to estimate parametric functions from the Kaplan–Meier curves in a Bayesian hierarchical model [19,20].

Statistical analyses were all performed within a Bayesian framework. Credible intervals were all reported at the 95% level. The contrast-based analysis was performed using R (version 3∙6∙0) (Core Team 2019, Vienna, Austria) and JAGS (version 4∙3∙0) with the package “getmtc” (version 0∙8∙2) [21] and Openbugs (version 3∙2∙3). Kaplan–Meier curves were reconstructed using GetData Graph Digitizer (version 2∙26).

### 2.3. Role of the Funding Source

There was no funding source for this study. The corresponding author had full access to all the data in the study and had final responsibility for the decision to submit for publication.

## 3. Results

**Figure 1:**
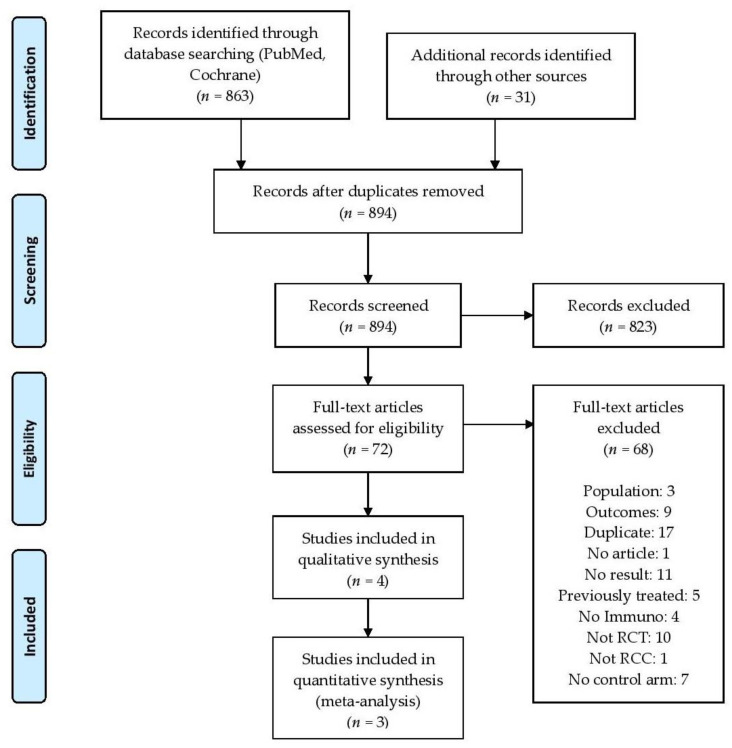
Flowchart of the systematic search. PRISMA flow diagram.

Our search identified 72 results. Of these, three published phase 3 randomized clinical trials matched our selection criteria (2843 patients, Figure 1): CheckMate 209–214 [4], Keynote 426 [8], and Javelin Renal 101 [9]. These evaluated three different combinations: nivolumab plus ipilimumab (Ipi-Nivo), pembrolizumab plus axitinib (Pembro-Axi), and avelumab plus axitinib (Ave-Axi), respectively (detailed search in File S1). The Immotion 151 trial investigating atezolizumab plus bevacizumab (Atezo-Beva) was excluded due to the non-superiority of OS compared to sunitinib in the intention-to-treat population. Therefore, it is unlikely that this combination will be recommended as a treatment in a near future. In the three retained trials, the combinations were compared to sunitinib (star-shaped network), which was the common comparator (trial characteristics are provided in Table 1). Risk of bias for each trial was considered acceptable in view of the Cochrane assessment grid (Table S1). Data sources for all the analyses are provided in Table S2.

### 3.1. Contrast-Based Approach in Intention-to-Treat Population

**Figure 2:**
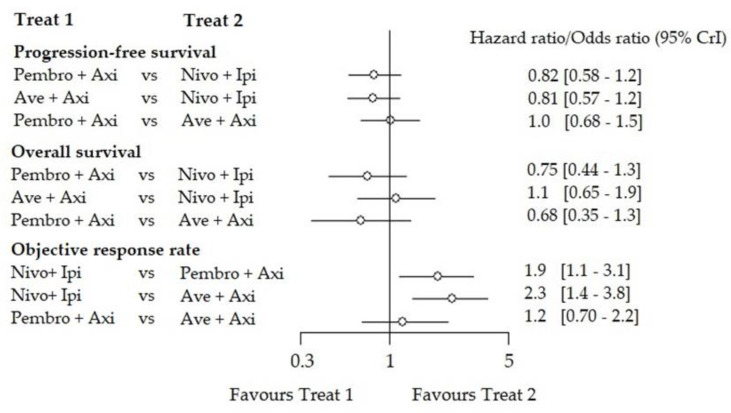
Indirect comparison of the contrast-based network meta-analysis (NMA) (fixed effect) in the ITT) population. Forest plot of the indirect comparison between each combination for the 3 outcomes in the ITT population. For the ORR, the odds ratio favoring treatment 1 Treat 1, means that treatment 1 had a lower response rate than treatment 2 (Treat 2).

Both Pembro-Axi and Ave-Axi showed similar efficacy for PFS (HR: 1.00 (0.68–1.50)). However, the Ipi-Nivo combination was less efficient (HR: 0.81 (0.57–1.20)) compared to Ave-Axi or Pembro-Axi (HR: 0.82 (0.58–1.20)). Ranking suggested Ave-Axi as the best option (SUCRA = 83%) and Pembro-Axi as the second best (SUCRA = 80%), but the difference was not clinically relevant (Table S3). For OS, NMA suggested Pembro-Axi (SUCRA = 96%) had better efficacy than Ave-Axi or Ipi-Nivo (HR: 0.68 (0.35–1.30), HR: 0.75 (0.44–1.30), respectively). Similarly, for ORR, NMA suggested that Ave-Axi (SUCRA = 94%) was the most preferable option compared to Pembro-Axi or Ipi-Nivo (odds ratio (OR) 0.81 (0.46–1.40), OR: 0.44 (0.27–0.72) respectively). These results are summarized in the form of forest plots for indirect comparisons (Figure 2) and direct comparisons (Figure S1) for the ITT population.

Fixed-effect and random-effect models yielded similar results, with larger credibility intervals for the random-effect model (Figure S2 and Table S3). Sensitivity analysis, either adding the fourth combination atezolizumab plus bevacizumab or using slightly informative priors, provided very close results with an unchanged rank order for the three combinations of the main analysis (Figure S3). 

### 3.2. Contrast-Based Approach per IMDC Subgroup

**Figure 3:**
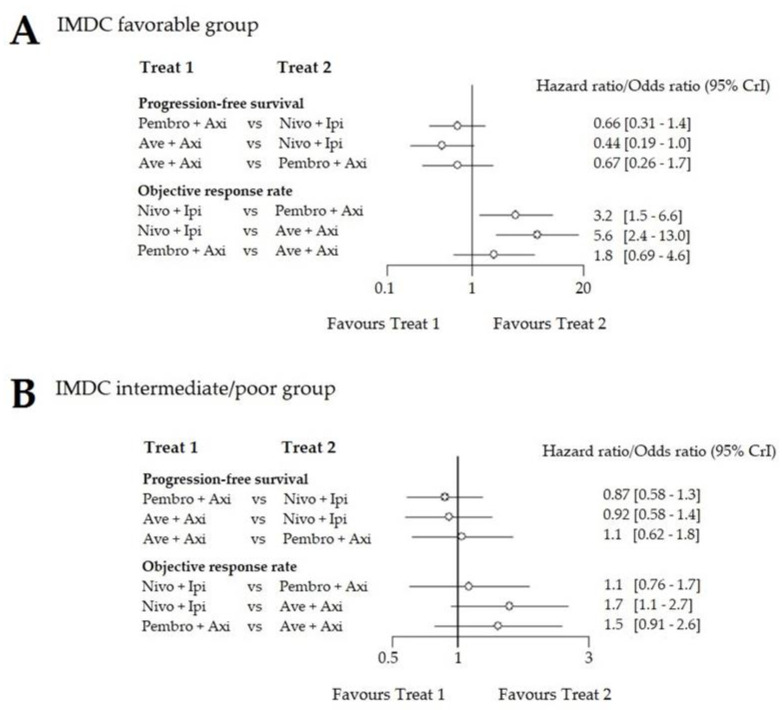
Forest plot of the indirect comparison between each combination. (**A**) results in the IMDC favorable risk group; (**B**) results in the IMDC intermediate and poor (pooled) risk group. For the For ORR, the odds ratio favouring treatment 1 (Treat 1) means that treatment 1 had a lower response rate than treatment 2 (Treat 2).

The IMDC subgroup analysis was performed for PFS and ORR only, since OS data were immature with many censored patients from the Javelin Renal 101 trial. We pooled the intermediate and poor IMDC risk subgroups to match the CheckMate 214 results with the other trials. Patient proportion in each subgroup is reported in Table 2. In the IMDC favorable risk group, Ave-Axi turned out to be superior to Pembro-Axi (HR for PFS: 0.67 (0.26–1.70), ORR: 1.8 (0.69–4.60)) and to Ipi-Nivo (HR for PFS: 0.44 (0.19–1.00), ORR: 5.6 (2.40–13.00)). In the intermediate and poor risk groups, Pembro-Axi and Ave-Axi were the two best options and compared favorably to Ipi-Nivo (Pembro-Axi HR for PFS: 0.87 (0.58–1.30), OR: 1.1 (0.76–1.70), Ave-Axi HR for PFS: 0.91 (0.58–1.40), OR: 1.7 (1.10–2.70)). The three combinations exhibited striking differences in the favorable risk group compared to the ITT analysis in terms of treatment effect, despite enlarged credibility intervals (Figure 3). Fixed-effect and random-effect models yielded similar results (ranking Table S4).

### 3.3. Arm-Based Approach

Among the different models tested, a Weibull model offered the best compromise between fit and complexity.

### 3.4. Progression-Free Survival in Intention-to-Treat Population

**Figure 4:**
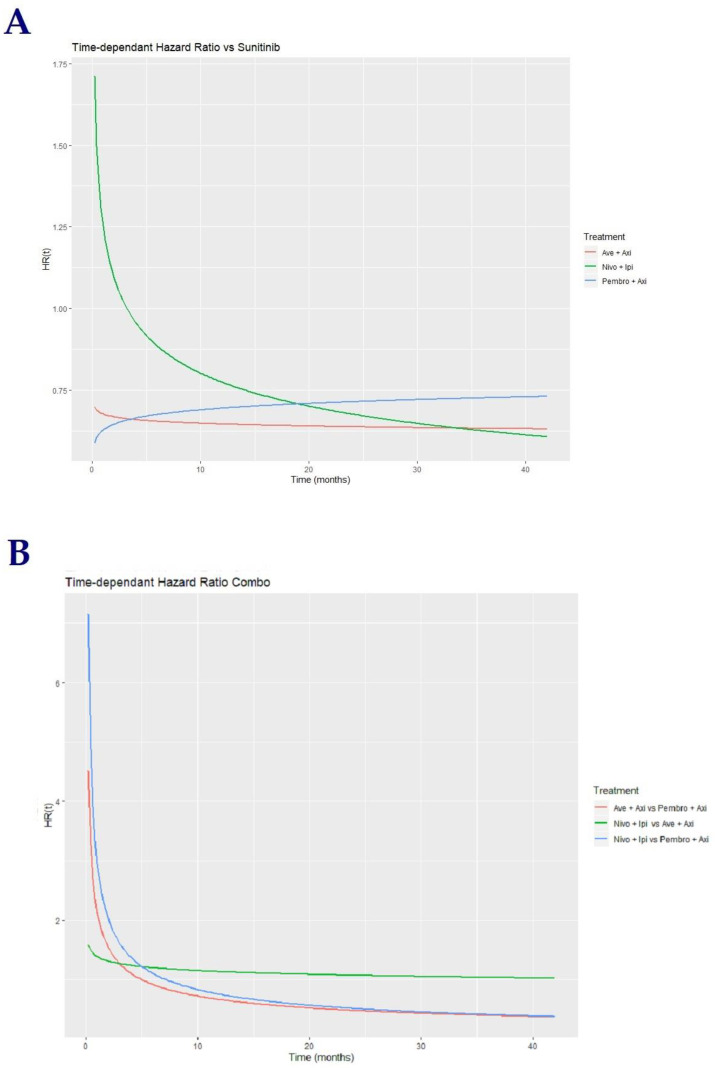
Time-dependent HRs for PFS of combinations. (**A**) Time-dependent hazard ratio vs. sunitinib. (**B**) Time-dependent hazard ratio between combinations.

The time-dependent HR of the drug combinations vs. sunitinib clearly suggest a violation of the main assumption of proportional hazards in the three trials, primarily for OS, and especially in the CheckMate 214 trial for both OS and PFS (Figure 4A). Risk of progression was higher with Ipi-Nivo compared to other combinations during the first 15 months; this difference vanished past this time period. Pembro-Axi and Ave-Axi exhibited close HR over the follow-up period; we considered that the seemingly different curves of time-dependent HR (increasing Pembro-Axi vs. decreasing Ave-Axi) were more a consequence of the models’ parameters than a real difference in the combinations’ effects (see parameter estimations, Table S5).

The aim of this study was to provide an indirect comparison between the three combinations. We allowed sunitinib’s effect to be different across each study instead of arbitrarily taking a mean effect, accounting for the variability of sunitinib’s effect, as observed in the different control arms. Benefit was in favor of Pembro-Axi over Ave-Axi and Ipi-Nivo during the first 5–7 months of treatment, which reversed afterwards. Ipi-Nivo and Ave-Axi displayed a comparable benefit with a higher risk of progression for Ipi-Nivo at the beginning of the treatment period, as in Figure 4B.

### 3.5. Overall Survival in Intention-to-Treat Population

**Figure 5:**
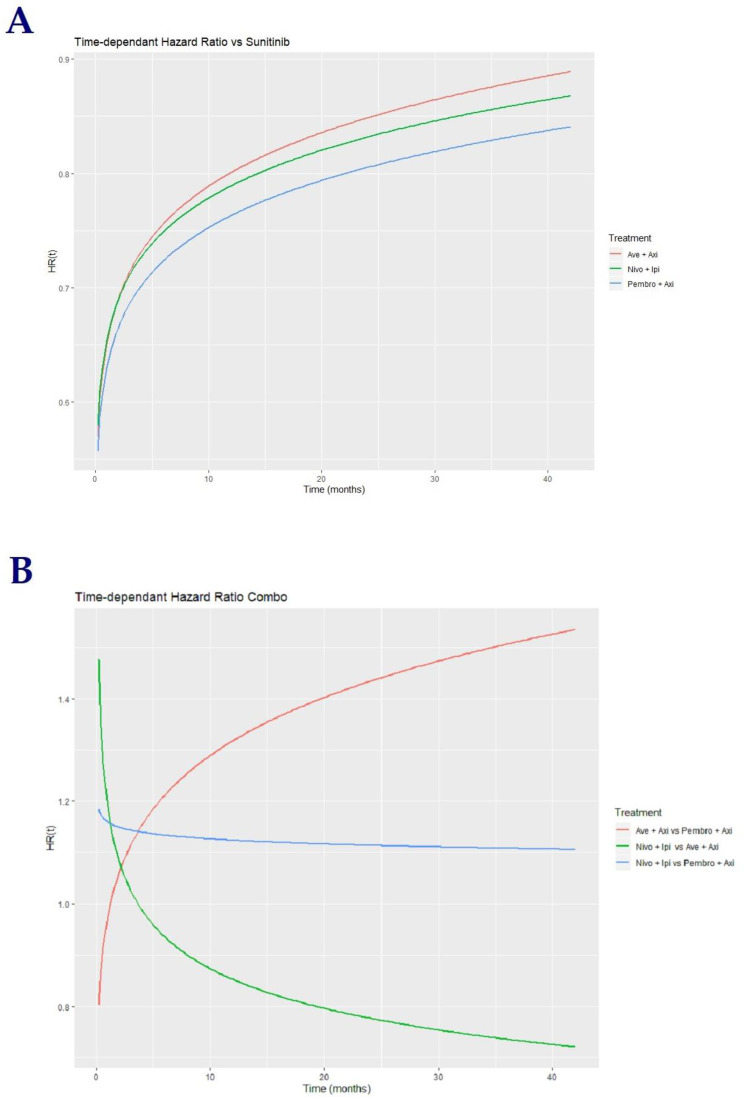
Time-dependent HRs for OS of combinations. (**A**) Time-dependent hazard ratio vs. sunitinib. Red: Ave-Axi vs. sunitinib, Green: Ipi-Nivo vs. sunitinib, Blue: Pembro-Axi vs. sunitinib. (**B**) Time-dependent hazard ratio between combinations. Red: Ave-Axi vs. Pembro-Axi, Green: Ipi-Nivo vs. Ave-Axi, Blue: Ipi-Nivo vs. Pembro-Axi.

The time-dependent HR curves for OS suggested that all three drug combinations have comparable time effects on OS (Figure 5A). We also showed that for each trial, the computed mean HR across the follow-up period exhibited fairly similar estimates, as in the contrast-based approach, and close to published HRs, which established the coherence between the two methods and conferred robustness to our results.

The main observations resulting from the indirect pairwise comparison of the three combinations suggested a higher risk of death with Ipi-Nivo compared to Pembro-Axi throughout the study period (Figure 5B). The higher risk of death of Ipi-Nivo compared to Ave-Axi was only observed during the first 3 months, which decreased afterwards. Pembro-Axi appeared as a better option compared to Ave-Axi during the whole follow-up period (see parameter estimations, Table S5).

### 3.6. Exploratory Analysis of PFS in Sarcomatoid Patients

**Figure 6:**
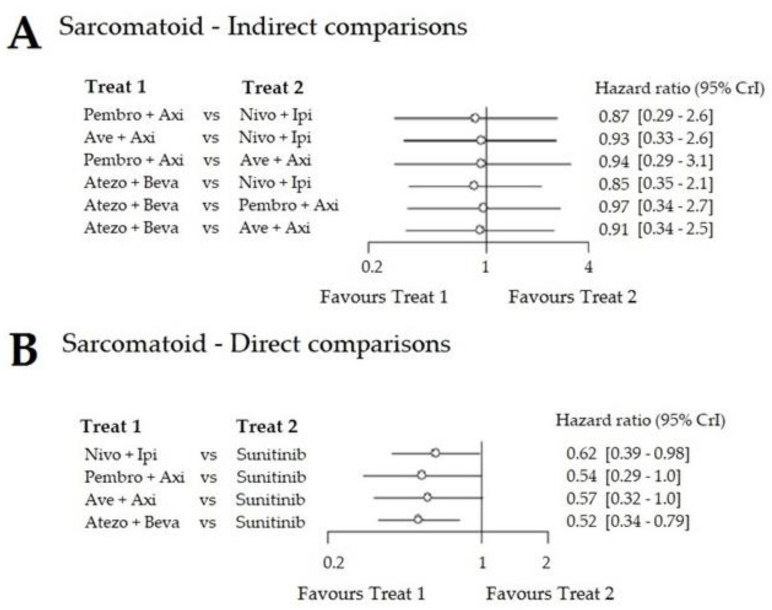
Forest plot of PFS in the sarcomatoid carcinoma population. (**A**) Direct comparisons. (**B**) Indirect comparisons. HRs of treatment in column A versus treatment in column B.

Upon indirect comparison, there was no significant difference between the trials, suggesting that these patients may respond well to all combinations (Figure 6), with all HRs close to 1.

## 4. Discussion

The three combinations considered in this study may soon become new standards of care in the first-line setting for mRCC, without any clear rationale to prefer one over the other. We aimed to fill this knowledge gap by conducting indirect comparisons, and thus hopefully providing clinicians with critical aid in decision-making.

Network meta-analysis is a powerful and flexible method of comparing multiple different therapeutic strategies. To our knowledge, few NMAs have been published in an mRCC first-line setting. Andrew W. Hahn et al. concluded that cabozantinib, Pembro-Axi, and Ave-Axi were preferable for PFS, and Pembro-Axi appeared superior for OS in first-line mRCC [22]. However, their network included twelve different treatments and highly heterogeneous populations. A recent study by Wang et al. included all available first-line options representing no less than twenty-five heterogeneous studies, and concluded that Pembro-Axi was the preferred option with regards to OS, whereas cabozantinib was better with regards to PFS [23]. However, in the last study included, HRs were compared assuming sunitinib had the same effect across all the different trials, which did not reflect actual/observed data/results.

However, our approach significantly differed from these studies: we specifically focused our comparison on the efficacies of the three combinations with immune checkpoint inhibitors that have demonstrated a benefit in phase 3 trials (i.e., the drug combinations more likely to obtain a high-grade recommendation from academic societies and an approval from health authorities). We used the most recent data (up to August 2019) and employed both fixed- and random-effect models. Moreover, we used two different approaches to assess these different therapeutic options: a contrast-based and an arm-based approach. In our arm-based approach, we relaxed the assumption of a common effect of sunitinib to best model the actual trial differences. We also investigated models that may have taken into account various confounding factors, such as the between-study unbalanced prognostic risk groups, in order to allow for sufficient flexibility in the modelling of these new combinations complexities (e.g., by adding a covariance term to model the presence of axitinib in both CPI-TKI combinations, and/or to combine PFS and OS in a single model). 

In our study, we compared three large multicenter phase 3 randomized controlled trials which included 2843 patients in total. In the contrast-based approach, Pembro-Axi was found to be the best option for the OS rate in the ITT population, whereas Pembro-Axi and Ave-Axi showed comparable efficacy for PFS, and Ave-Axi showed the best ORR efficacy. On the other hand, in the IMDC favorable risk group, Ave-Axi showed the most favorable results for PFS. Contrast-based approaches for both PFS and OS led to results close to what was reported in each independent updated study, due to the fact that only one study was available for each comparison and that we decided upon non-informative priors for treatment effects (i.e., no influencing data). In the arm-based approach (in the ITT population), Pembro-Axi seemed to be the preferable option only for the OS. We also observed that during the first 5 months of therapy, IO-TKI (immunotherapy and TKI) combinations exhibited a lower risk of progression compared with IO-IO combinations; however, Ipi-Nivo exhibited longer PFS in patients who did not progress during the first 5 months. This may be partially related to pseudo progression induced by the double IO combination, while having a high rate of complete response for the remaining patients in the CheckMate 214 trial.

Our study has both strengths and limitations. First, we focused on the new promising regimens, with results from published phase 3 randomized clinical trials, in order not to inflate population and design heterogeneity. Second, we used two different but complementary approaches for more consistent results: the contrast-based approach, which uses HRs as relative treatment effects and maintains the randomization structure within each study but requires strong assumptions. An arm-based approach is more likely to relax these assumptions, but the disadvantage is that it does not preserve the randomization structure. Moreover, in the contrast-based approach, HRs derived from the Cox model rely on the assumption of proportional hazards, which is commonly violated in many trials, leading to biased estimates. Arm-based methods do not rely on HR but need the parametric fit of Kaplan–Meier (KM) curves. Third, combinations may have more complex mechanisms of action than monotherapies, and to this end arm-based methods provide time-dependent HR, interpretations of which may help to decipher such mechanisms better than constant HR and decide which combination may be the best and when. One main limitation is the overall lack of data, which may reflect a potential uncontrolled bias; more studies comparing these regimens and/or individual patient data would be needed in order to improve the precision and heterogeneity of estimations. These additional data would also allow us to test for inconsistency (confirm concordance between direct and indirect comparisons), which was not possible in our current star-shaped network. IMDC subgroups and geographic regions may represent other confounding factors across comparisons; more studies are needed to adjust the NMA model and confirm our findings. We tried more complex multivariate NMAs to account for HRs per IMDC subgroup in one single model, but a lack of data for OS in each risk group prevented us from refining the final model. It could also have been relevant to consider PD-L1 expression, which may have differently influenced the PFS of the combinations, but given the different assays and thresholds used in each study, we could not proceed. Regarding toxicity, NMA using only counts of grade ≥ 3 events was too broad to efficiently compare toxicity between trials. Lastly, OS and possibly ORR data in the Javelin Renal 101 trial were still immature at the time of analysis; thus, the Ave-Axi combination ranking may change with a longer follow-up. Despite a comparable median follow-up, Pembro-Axi exhibited superiority in terms of OS, whereas Ave-Axi surprisingly did not. Our indirect comparison was indeed in favor of Pembro-Axi, but more updates and trials would be needed to further investigate this difference. Therefore, the results of our study should be interpreted cautiously given the underlying hypothesis and potential bias of the estimated effects.

Clinicians have concerns about sequencing and identifying predictive biomarkers. More follow-up and reported data from patients in second-line after IO-TKI and IO-IO combination treatments may be of great help to guide decisions about the line of treatment. Our NMA model can grow with each new trial to help decision-making. Other trial results are awaited, comparing pembrolizumab plus lenvatinib vs. everolimus plus lenvatinib vs. sunitinib (CLEAR, NCT02811861); triplet cabozantinib plus nivolumab plus ipilimumab vs. nivolumab plus ipilimumab (COSMIC-313, NCT03937219); and nivolumab plus cabozantinib vs. cabozantinib plus nivolumab plus ipilimumab vs. sunitinib (CheckMate 9ER, NCT03141177). Personalized-therapy-driven trials based on molecular profiling such as the BIONIKK trial (NCT02960906) may also provide new insights for clinical decision.

## 5. Conclusions

Our results support the importance of the IMDC risk score for the comparative efficacy assessment of new combinations in the first-line setting of metastatic clear-cell renal cell carcinoma. This is important given the lack of predictive validated biomarkers. Our results suggest a PFS, ORR, and OS superiority of IO-TKI, compared with IO-IO combinations, regardless of the IMDC risk group. In favorable risk-group patients, PFS and OS were superior with IO-TKI, but these differences vanished in the intermediate/poor risk group. Overall, based on the current evidence, pembrolizumab-axitinib may have a slight advantage over the two other combinations.

## Figures and Tables

**Table 1:** Outcomes reported in each trial of the network. Ave: avelumab; Axi: axitinib; Ipi: ipilimumab; Nivo: nivolumab; NR: not reached; ORR: objective response rate; OS: overall survival; Pembro: pembrolizumab; PFS: progression-free survival.

Study	Treatment	Number of Patients	ORR (95% CI)	Median OS (Months)	Median PFS (Months)	HR OS (95% CI)	HR PFS (95% CI)
**CheckMate 214**	Sunitinib	546	32% (28–36)	37.9	12.3	-	-
	Nivo + Ipi	550	39% (35–43)	NR	12.4	0.71 (0.59–0.86)	0.85 (0.73–0.98)
**Keynote 426**	Sunitinib	429	35.7% (31–40)	NR	11.1	-	-
	Pembro + Axi	432	59% (54–64)	NR	15.1	0.53 (0.38–0.74)	0.69 (0.57–0.84)
**Javelin Renal 101**	Sunitinib	444	25% (22–30)	NR	8.4	-	-
	Ave + Axi	442	51% (47–56)	NR	13.8	0.78 (0.55–1.08)	0.69 (0.56–0.84)

**Table 2:** Summary data in each IMDC subgroup.

Trial	Treatment	Favorable Prognosis			Intermediate and Poor Prognosis		
		N (%)	HR IC 95%	ORR IC 95%	N (%)	HR IC 95%	ORR IC 95%
**CheckMate 214**	Sunitinib	124 (23)		50%	424 (77)		29%
	Nivo + Ipi	125 (23)	1.23 (0.90–1.69)	39%	423 (77)	0.77 (0.65–0.90)	42%
**Keynote 426**	Sunitinib	131 (31)		49.6%	298 (69)		29.5%
	Pembro + Axi	138 (32)	0.81 (0.53–1.24)	66.7%	294 (68)	0.67 (0.53–0.85)	55.8%
**Javelin Renal 101**	Sunitinib	96 (22)		37%	347 (78)		22.5%
	Ave + Axi	94 (22)	0.54 (0.32–0.91)	68.1%	343 (78)	0.70 (0.53–0.94)	46.9%

Note: the sum of patients in the (reported) subgroup analysis was different from the overall number of patients reported in the articles.

## Supplementary Materials

The following are available online at https://www.mdpi.com/2072-6694/12/6/1673/s1: Figure S1: Forest plot for direct comparisons in the ITT population, Figure S2: Forest plot of the indirect comparison between each combination for the 3 outcomes in the ITT population using a random effect model with informative priors, Figure S3: Forest plot of including Atezo + Beva as sensitivity analysis in the ITT population, Table S1: Bias assessment risk for the 3 selected studies of the network, Table S2: Sources of all data extracted, Table S3: Ranking of PFS, OS and ORR in the ITT population, contrast-based approach, Table S4: Ranking of PFS and ORR in the IMDC good and intermediate/poor (pooled) risk groups, contrast-based approach, Table S5: Parameter estimation of the arm-based Weibull model for PFS and OS, File S1: Detailed search strategy for systematic review.
