# Supplementary material for: Comparative Efficacy of First-Line Immune-Based Combination Therapies in Metastatic Renal Cell Carcinoma: A Systematic Review and Network Meta-Analysis
Source: Cancers (Basel). 2020 Jun 24;12(6):1673. doi: 10.3390/cancers12061673 (PMC7352474; doi:10.3390/cancers12061673)
Supplement: Supplementary file 1 [file cancers-12-01673-s001.pdf]

Supplementary Materials

Comparative Efficacy of First-Line Immune-Based Combination Therapies in Metastatic Renal Cell Carcinoma: A Systematic Review and Network Meta-Analysis

Reza Elaidi, Letuan Phan, Delphine Borchiellini, Philippe Barthelemy, Alain Ravaud, Stéphane Oudard and Yann Vano

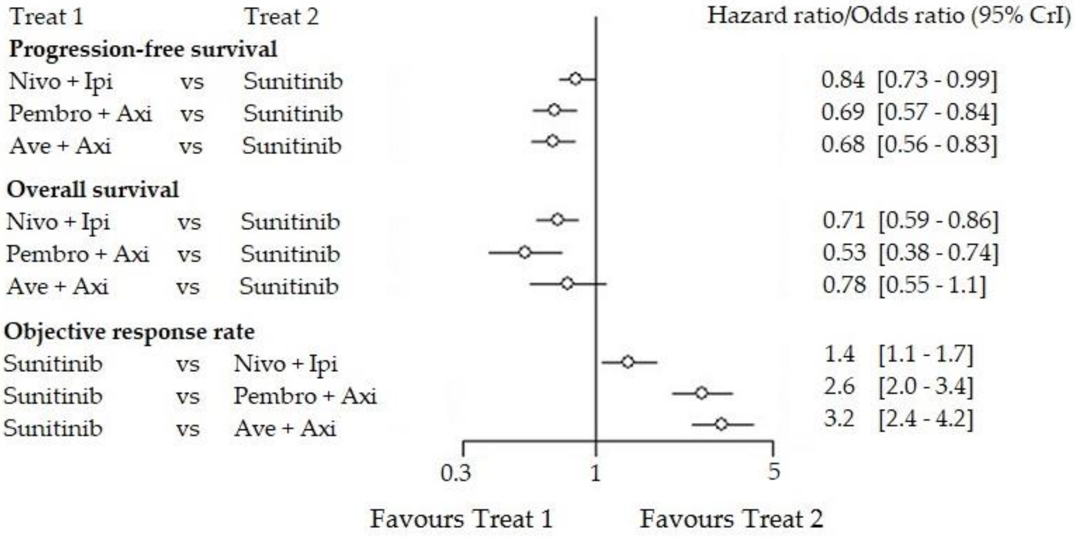

Figure S1. Forest plot for direct comparisons in the ITT population. HRs and ORs compare treatment 1 (Treat 1) to treatment 2 (Treat 2) (Online only).

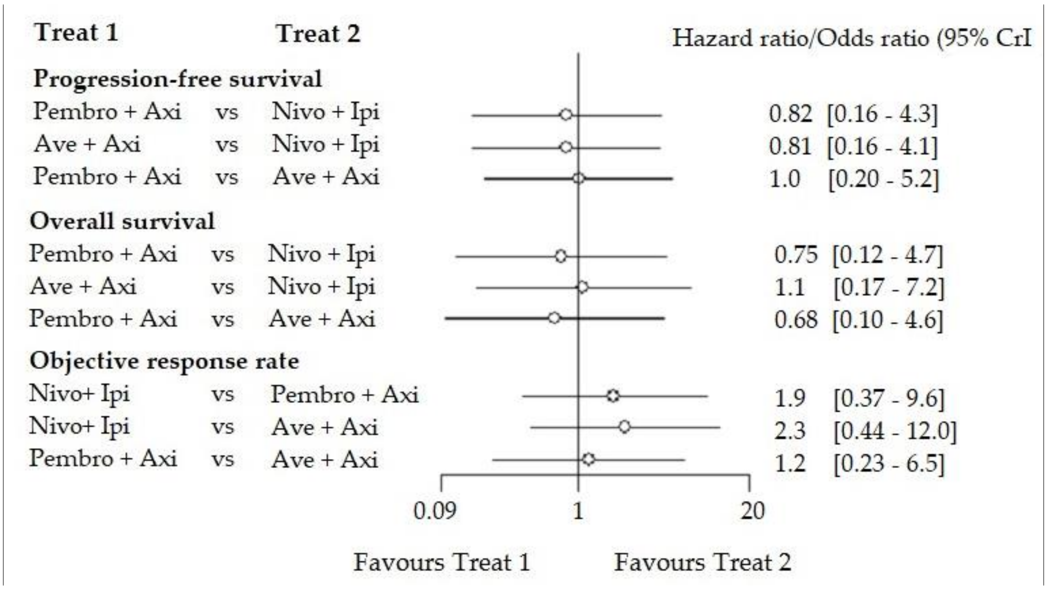

Figure S2. Forest plot of the indirect comparison between each combination for the 3 outcomes in the ITT population using a random effect model with informative priors (Online only). HRs and ORs compare treatment 1 (Treat 1) to treatment 2 (Treat 2). We used empirical priors for the between-study variance (heterogeneity) as proposed by R.Turner (2015). Other priors were also

tested as sensitivity analysis, but very few information being available for the combinations, more data would be needed to build more reliable informative priors.

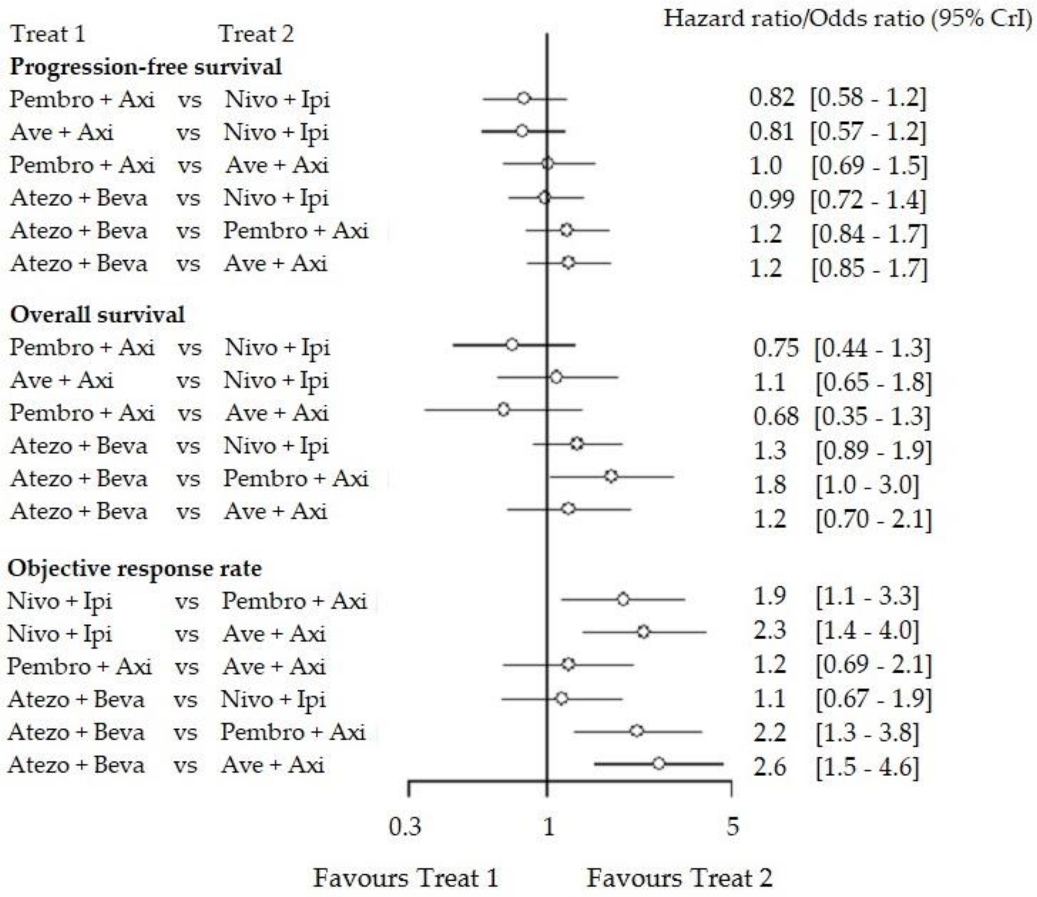

Figure S3. Forest plot of including Atezo + Beva as sensitivity analysis in the ITT population. HRs and ORs compare treatment 1 (Treat 1) to treatment 2 (Treat 2) (Online only).

Table S1. Bias assessment risk for the 3 selected studies of the network (Online only).

Bias source	Checkmate 214	Keynote 426	Javelin Renal 101
**Random Sequence Generation**	Low	Low	Low
**Allocation Concealment**	Low	Low	Low
**Blinding of Participants and Personnel**	High	High	High
**Blinding (Outcome Assessment)**	Unclear	Low	Low
**Incomplete Outcome Data**	Low	Low	Unclear
**Selective Reporting**	Low	Low	Low
**Other Sources of Bias**	Low	Low	Low

Table S2. Sources of all data extracted (Online only).

Population	Trial	Outcome	Source
ITT	Checkmate 214	PFS	Article–The Lancet
		OS	Article–The Lancet
		ORR	Article–The Lancet
	Keynote 426	PFS	Article–New England Journal of Medicine
		OS	Article–New England Journal of Medicine
		ORR	Article–New England Journal of Medicine
	Javelin renal 101	PFS	Presentation at ASCO GU
		OS	Article–New England Journal of Medicine
		ORR	Presentation at ASCO GU
	IMmotion 151	PFS	Article–The Lancet
		OS	Article–The Lancet
		ORR	Article–The Lancet
IMDC Subgroups	Checkmate 214	PFS	Article–The Lancet
		ORR	Article–The Lancet
	Keynote 426	PFS	Presentation at ASCO
		ORR	Presentation at ASCO
	Javelin renal 101	PFS	Presentation at ASCO GU
		ORR	Presentation at ASCO GU
	CABOSUN	PFS	Article–European Journal of Cancer
		ORR	Article–European Journal of Cancer

Table 3. Ranking of PFS, OS and ORR in the ITT population, contrast-based approach (Online only).

**Progression-Free Survival**					
**Fixed Effect Model**	**Rank 1**	**Rank 2**	**Rank 3**	**Rank 4**	**SUCRA**
Nivo + Ipi	0.011	0.089	0.884	0.017	37%
Pembro + Axi	0.466	0.480	0.053	0.000	80%
Ave + Axi	0.523	0.431	0.046	0.000	83%
Sunitinib	0.000	0.000	0.017	0.983	1%
**Random Effect Model**					
Nivo + Ipi	0.132	0.2	0.468	0.171	42%
Pembro + Axi	0.411	0.362	0.146	0.081	70%
Ave + Axi	0.447	0.340	0.140	0.073	72%
Sunitinib	0.010	0.069	0.247	0.675	14%
**Overall Survival**					
**Fixed Effect Model**	**Rank 1**	**Rank 2**	**Rank 3**	**Rank 4**	**SUCRA**
Nivo + Ipi	0.058	0.613	0.329	0.000	58%
Pembro + Axi	0.892	0.085	0.023	0.000	96%
Ave + Axi	0.050	0.302	0.584	0.064	45%
Sunitinib	0.000	0.000	0.064	0.936	2%
**Random Effect Model**					
Nivo + Ipi	0.122	0.527	0.306	0.045	58%
Pembro + Axi	0.784	0.155	0.052	0.010	90%
Ave + Axi	0.094	0.299	0.459	0.148	45%
Sunitinib	0.000	0.019	0.183	0.797	7%
**Objective Response Rate**					
**Fixed Effect Model**	**Rank 1**	**Rank 2**	**Rank 3**	**Rank 4**	**SUCRA**
Nivo + Ipi	0.000	0.000	0.993	0.007	33%
Pembro + Axi	0.174	0.826	0.000	0.000	73%
Ave + Axi	0.826	0.174	0.000	0.000	94%
Sunitinib	0.000	0.000	0.007	0.993	2%
**Random Effect Model**					
Nivo + Ipi	0.028	0.073	0.767	0.132	58%
Pembro + Axi	0.286	0.641	0.055	0.018	73%
Ave + Axi	0.684	0.272	0.033	0.010	88%
Sunitinib	0.002	0.013	0.145	0.840	6%

Table 4. Ranking of PFS and ORR in the IMDC good and intermediate/poor (pooled) risk groups, contrast-based approach (Online only).

Progression-Free Survival					
**IMDC Good Prognosis Population**					
**Fixed Effect Model**	**Rank 1**	**Rank 2**	**Rank 3**	**Rank 4**	**SUCRA**
Nivo + Ipi	0.001	0.025	0.096	0.878	5%
Pembro + Axi	0.125	0.703	0.124	0.048	63%
Ave + Axi	0.873	0.117	0.008	0.003	95%
Sunitinib	0.002	0.155	0.772	0.071	36%
**Random Effect Model**	**Rank 1**	**Rank 2**	**Rank 3**	**Rank 4**	**SUCRA**
Nivo + Ipi	0.039	0.100	0.176	0.685	16%
Pembro + Axi	0.193	0.520	0.166	0.121	60%
Ave + Axi	0.752	0.174	0.042	0.032	88%
Sunitinib	0.016	0.206	0.616	0.162	36%
Nivo + Ipi	0.001	0.025	0.096	0.878	5%
**IMDC Intermediate/Poor Prognosis Population**					
**Fixed Effect Model**	**Rank 1**	**Rank 2**	**Rank 3**	**Rank 4**	**SUCRA**
Nivo + Ipi	0.087	0.310	0.602	0.001	49%
Pembro + Axi	0.553	0.329	0.118	0.000	81%
Ave + Axi	0.360	0.361	0.270	0.009	69%
Sunitinib	0.000	0.000	0.010	0.990	3%
**Random Effect Model**	**Rank 1**	**Rank 2**	**Rank 3**	**Rank 4**	**SUCRA**
Nivo + Ipi	0.188	0.311	0.379	0.121	52%
Pembro + Axi	0.459	0.304	0.167	0.069	72%
Ave + Axi	0.344	0.325	0.234	0.097	64%
Sunitinib	0.009	0.059	0.220	0.712	12%
**Objective Response Rate**					
**IMDC Good Prognosis Population**					
**Fixed Effect Model**	**Rank 1**	**Rank 2**	**Rank 3**	**Rank 4**	**SUCRA**
Nivo + Ipi	0.000	0.001	0.040	0.96	1%
Pembro + Axi	0.076	0.923	0.002	0.00	69%
Ave + Axi	0.924	0.076	0.000	0.00	97%
Sunitinib	0.000	0.001	0.959	0.04	32%
**Random Effect Model**	**Rank 1**	**Rank 2**	**Rank 3**	**Rank 4**	**SUCRA**
Nivo + Ipi	0.062	0.116	0.170	0.653	20%
Pembro + Axi	0.272	0.475	0.135	0.119	63%
Ave + Axi	0.645	0.226	0.080	0.049	82%
Sunitinib	0.021	0.184	0.616	0.179	35%
**IMDC Intermediate/Poor Prognosis Population**					
**Fixed Effect Model**	**Rank 1**	**Rank 2**	**Rank 3**	**Rank 4**	**SUCRA**
Nivo + Ipi	0.005	0.295	0.700	0	44%
Pembro + Axi	0.040	0.661	0.299	0	58%
Ave + Axi	0.955	0.044	0.001	0	98%
Sunitinib	0.000	0.000	0.000	1	0%
**Random Effect Model**	**Rank 1**	**Rank 2**	**Rank 3**	**Rank 4**	**SUCRA**
Nivo + Ipi	0.198	0.293	0.322	0.187	50%
Pembro + Axi	0.249	0.341	0.246	0.164	56%
Ave + Axi	0.541	0.257	0.120	0.082	75%
Sunitinib	0.012	0.110	0.311	0.567	19%

Table S5. Parameter estimation of the arm-based Weibull model for PFS and OS (Online only).

Study	Parameter	Estimation	SD	95% CrI<br>CrI upper
**Progression-Free Survival**				
**Checkmate 214 Nivo + Ipi**	Delta1	0.2343	0.1018	(−0.422; −0.0177)
	Delta2	−0.094	0.1074	(−0.314; 0.110)
	Mu1	−2.248	0.0825	(−2.415; −2.091)
	Mu2	−0.4521	0.0902	(−0.6281; −0.275)
**Keynote 426 Pembro + Axi**	Delta1	−0.2866	0.0996	(−0.494; −0.101)
	Delta2	−0.0563	0.1166	(−0.270; 0.189)
	Mu1	−2.752	0.0878	(−2.925; −2.581)
	Mu2	−0.0140	0.1265	(−0.264; 0.232)
**Javelin Renal 101 Ave + Axi**	Delta1	−0.2888	0.101	(−0.498; −0.0996)
	Delta2	−0.071	0.1049	(−0.272; 0.140)
	Mu1	−2.336	0.0916	(−2.517; −2.158)
	Mu2	−0.241	0.09381	(−0.426; −0.058)
**Overall Survival**				
**Checkmate 214 Nivo + Ipi**	Delta1	−0.424	0.159	(−0.730; −0.108)
	Delta2	0.076	0.107	(−0.137; 0.282)
	Mu1	−4.001	0.129	(−4.257; −3.752)
	Mu2	0.056	0.083	(−0.108; 0.220)
**Keynote 426 Pembro + Axi**	Delta1	0.076	0.107	(−0.137; 0.282)
	Delta2	−0.462	0.162	(−0.787; −0.150)
	Mu1	−4.112	0.153	(−4.421; −3.821)
	Mu2	0.068	0.177	(−0.277; 0.411)
**Javelin Renal 101 Ave + Axi**	Delta1	0.077	0.130	(−0.175; 0.334)
	Delta2	−0.429	0.164	(−0.748; −0.106)
	Mu1	−4.170	0.182	(−4.537; −3.824)
	Mu2	0.183	0.133	(−0.078; 0.443)

Note: Delta 1 and Delta 2 refer to the experimental arm Weibull parameters, as described by Ouwen and Jansen. Mu 1 and Mu 2 refer to the control arm Weibull parameters, as described by Ouwen and Jansen.

File S1: Detailed search strategy for systematic review (Online only)

Research question: Comparing the efficacy of the new treatment combinations for patients with metastatic renal cell carcinoma in the first-line setting, in term of progression-free survival, overall survival and objective response rate.

Eligibility criteria

Inclusion criteria:

Randomized controlled trial
Include patients with metastatic renal cell carcinoma
Experimental treatment involves immune checkpoint inhibitor combination

Exclusion criteria:

Not reporting the co-primary outcomes
Include only subpopulation (i.e. age or region restraint, IMDC subgroups etc.)
Include patients previously treated for renal cell carcinoma (not in first-line setting)

Searched databases:

Pubmed, Cochrane library, Clinicaltrial.gov, relevant congresses (ESMO, ASCO, etc.)

Keyword:

"renal cell carcinoma" AND (randomized controlled trial)

Details in PubMed:

"renal cell carcinoma"[All Fields] AND ("randomized controlled trial"[Publication Type] OR "randomized controlled trials as topic"[MeSH Terms] OR "randomized controlled trial"[All Fields] OR "randomised controlled trial"[All Fields]) AND ("2015/01/01"[PDAT] : "2019/10/31"[PDAT])

Cochrane Library:

(randomized clinical trial "renal cell carcinoma" metastatic or advanced "Immune checkpoint inhibitor"):ti,ab,kw with Publication Year from 2015 to 2019, with Cochrane Library publication date Between Jan 2015 and Oct 2019, in Trials (Word variations have been searched)
(See flowchart for election process)

© 2020 by the authors. Licensee MDPI, Basel, Switzerland. This article is an open access article distributed under the terms and conditions of the Creative Commons Attribution (CC BY) license (http://creativecommons.org/licenses/by/4.0/).
